# Standardizing quantitative muscle MRI: an inter-vendor evaluation in healthy adults

**DOI:** 10.1007/s10334-026-01328-9

**Published:** 2026-02-12

**Authors:** Johanna Thomä, Lionel Butry, Martijn Froeling, Marco Meixner, Johannes Forsting, Lara Schlaffke

**Affiliations:** 1 Department of Neurology, BG-University Hospital Bergmannsheil, Ruhr-University Bochum, 44789 Bochum, Germany; 2 Heimer Institute for Muscle Research, BG-University Hospital Bergmannsheil, Ruhr-University Bochum, 44789 Bochum, Germany; 3 Center for Image Sciences, Precision Imaging Group, Division Imaging & Oncology, University Medical Centre Utrecht, Utrecht, Netherlands; 4 Institute of Neuroradiology, St. Josef Hospital, Ruhr-University Bochum, 44791 Bochum, Germany; 5 Dep. Information Technology, University of Applied Sciences and Arts, 44139 Dortmund, Germany; 6 University Hospital of Pediatrics and Adolescent Medicine, Ruhr-University Bochum, 44791 Bochum, Germany; 7 Department of Neurology, St. Josef Hospital, Ruhr-University Bochum, 44791 Bochum, Germany

**Keywords:** Magnet resonance imaging, Muscle tissue, Medical imaging, Reproducibility of results

## Abstract

**Objectives:**

Quantitative muscle MRI (qMRI) is applied to detect muscular alterations, particularly in neuromuscular diseases (NMDs). As studies on rare NMDs benefit from multi-center designs, inter-vendor reproducibility must be assessed to ensure data comparability. However, manufacturer-related variability has been limited investigated. This study evaluates inter-vendor reproducibility of qMRI parameters in healthy subjects.

**Methods:**

Ten healthy volunteers were investigated within 1 week on two 3T MRI systems (Siemens, Philips). Twelve leg muscles were analyzed using Dixon (fat fraction, FF), T2 mapping (water T2, wT2), and diffusion-weighted-sequence (diffusion parameters: fractional anisotropy (FA), mean, radial, axial diffusivity [MD, RD, *λ*_1_]). Reproducibility was assessed using intraclass correlation coefficient (ICC), within-subject coefficients of variation, *t* tests, and Bland–Altman analysis.

**Results:**

ICCs demonstrated moderate to excellent reproducibility for most parameters (ICC_FF_ = 0.923, ICC_wT2_ = 0.792, ICC_FA_ = 0.777, ICC_MD_ = 0.708, ICC_RD_ = 0.850), except for *λ*_1_ (ICC = 0.361). Significant inter-vendor differences were observed for all parameters (*p* < 0.05), with systematic biases ranging from 44.2% for FF to 3.6% for FA.

**Discussion:**

qMRI parameters were generally reproducible in healthy volunteers. However, absolute values differed between manufacturers and require correction. These differences should be considered to minimize manufacturer-related discrepancies and to facilitate data pooling in multi-center and longitudinal studies.

**Supplementary Information:**

The online version contains supplementary material available at 10.1007/s10334-026-01328-9.

## Introduction

Quantitative resonance imaging (qMRI) for muscle is a valuable and non-invasive tool in the evaluation of muscle disorders, including muscular injuries and neuromuscular diseases (NMDs) [1]. The availability of a large and comparable database is critical, especially for evaluating the efficacy of emerging therapies, such as gene therapies, in the context of rare NMDs.

Various qMRI metrics can be used to assess distinct muscle characteristics. Dixon-based sequences enable the assessment of fat fraction (FF) in individual muscles, allowing the quantification of fat replacement, as seen in various NMDs such as Becker and Duchenne muscular dystrophy [2, 3], Pompe disease [4], facioscapulohumeral muscle dystrophy (FSHD) [5] or inclusion body myositis [6]. Measuring the water T2 relaxation time (wT2) allows to visualize inflammation and muscle edema, as active muscle damage is associated with increased water content [7].

Diffusion tensor imaging (DTI) can assess micro- and macrostructural properties, providing insights into tissue integrity. This allows the early detection of pathologic changes in muscular injuries, inflammation, or NMDs [6, 8–10].

While these methods and their applications have been well documented and correlate with muscle biopsies [7, 11–13], achieving standardized acquisition and evaluation for consistent inter-vendor comparability remains challenging. Previous studies have demonstrated promising long-term reproducibility both in single-scanner datasets [14] and in multi-center studies, utilizing scanners from the same manufacturer [15]. Data on multi-vendor comparability are limited [16–19] and no study to date has systematically compared quantitative muscle MRI metrics across major MRI vendors using standardized analysis pipelines. The diversity of MR systems from various manufacturers presents a significant challenge for data pooling, which is crucial in studying rare diseases due to limited sample sizes. Therefore, the comparability of data across vendors is of particular importance for the integration of imaging biomarkers into clinical trials and for the establishment of large-scale normative databases. This comparability is especially crucial in multi-center studies aimed at the development and evaluation of therapeutic strategies and treatment-related outcomes. The matter is also relevant in clinical practice, where follow-up imaging can be performed using scanners from different vendors or after scanner upgrades.

Exploring the reproducibility of data acquired from scanners by different vendors can expand the knowledge base and ensure comparability across study sites. In the context of replacing a scanner at a single site—reflecting a real-life scenario—study used widely available sequences that were as standardized as possible to minimize site-specific differences. Therefore, this study aims to evaluate the reproducibility of the quantitative parameters FF, wT2, and diffusion metrics across two MRI vendors (Philips and Siemens) in a traveling cohort.

## Methods

### Study design and population

A total of ten healthy participants aged 24–34 years were recruited from the local community, with equal representation of both sexes (5 females, 5 males). Each participant underwent assessments of 12 thigh muscles using two 3T MRI scanners, one from Philips and one from Siemens, within 1 week to minimize the risk of physiological changes affecting the results. Information regarding unusual physical activities, the mode of transportation to the study site (bicycle, walking, public transport, or car), and the travel duration was collected. Exclusion criteria included any muscular or neurological disorders. The ethics committee of Ruhr-University Bochum approved the study (No 15-5281, 29 January 2021). All participants provided written informed consent.

### MRI data acquisition

**Fig. 1:**
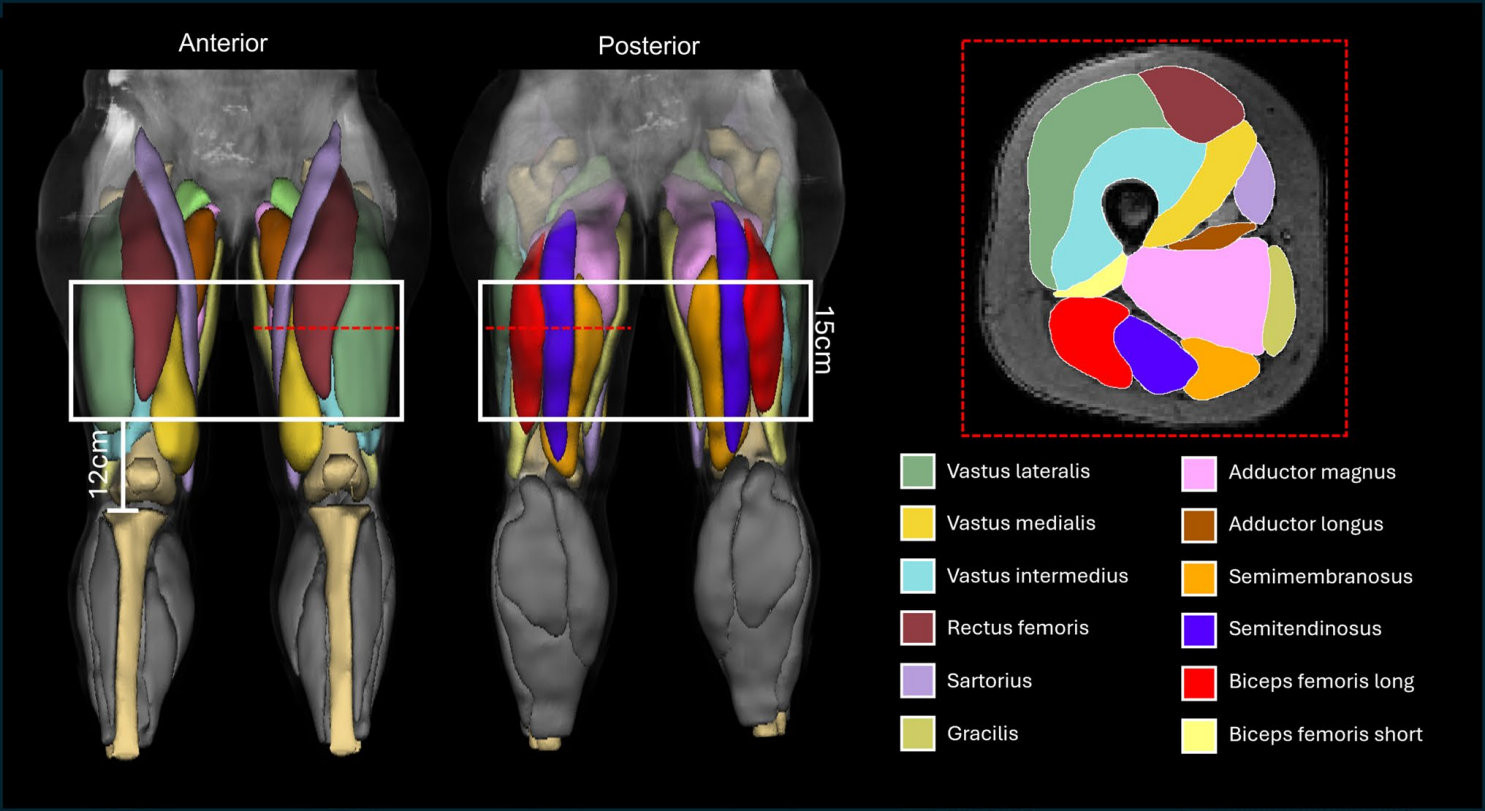
Position settings for the field of view (FOV) and muscle segmentation of the 12 analyzed thigh muscles

The participants were positioned feet-first in the scanner, with their feet secured using sandbags to minimize movement. No specific resting period was applied prior to scanning. The examinations were conducted using a Siemens Magnetom Prisma with an 18-channel body coil and a Philips Achieva 3.0-T MRI with a 16-channel body coil. The field of view (FOV) was uniformly set 120 mm proximal to the tibial plateau, with dimensions of 480 × 276 × 150 mm^3^ on the Philips scanner and 480 × 390 (qT2)/282 (DWI)/315 (Dixon) × 150 on the Siemens scanner (Fig. 1). The scanning protocol included a four-point (Philips) and six-point (Siemens) Dixon-based sequence for fat fraction assessment, a multi-echo spin-echo sequence (MESE) for quantitative water mapping with sinc refocusing pulses, and a diffusion-weighted spin-echo echo-planar sequence (SE-EPI). Detailed information is provided in Table 1.

**Table 1:** Scan parameter: parameters for quantitative T2 (qT2), diffusion-weighted imaging (DWI) and Dixon sequences for Philips and Siemens

	**qT2**	**DWI**	**Dixon**
Sequence	MESE	SE-EPI	MS_FFE, 3D-Aquisition (Siemens)
FOV (LR x AP xFH mm^3^)			
Philips	480 × 276x150	480 × 276x150	480 × 276x150
Siemens	480 × 390x150	480 × 282x150	480 × 315x150
Acquisition matrix			
Philips	160 × 92	160 × 92	320 × 184
Siemens	160 × 130	160 × 94	320 × 210
Voxel size (mm^3^)			
Philips	3 × 3x6	3 × 3x6	1.5 × 1.5x6
Siemens	3 × 3x6	3 × 3x6	1.5 × 1.5x5
Slice gap (mm)			
Philips	6	–	–
Siemens	6	–	1
Slices			
Philips	13	25	25
Siemens	13	25	30
TR (ms)			
Philips	7650	5000	210
Siemens	4600	5600	15
TE (ms)			
Philips	17 × Δ 7.6	57	2.6/3.36/4.12/4.88
Siemens	17 × Δ 7.6	60.2	1.46/3/4.54/6.08/7.62/9.16
Flip angle (°)			
Philips	90/180		8
Siemens	90/180		5
Parallel acquisition techniques (acceleration factor)			
Philips	SENSE (2)	SENSE (1.9)	SENSE (2)
Siemens	GRAPPA (2)	GRAPPA (4)	CAIPIRINHA (2)
b values (number of images) Philips Siemens	–	0 (1), 1 (6), 10 (3), 25 (3), 100 (3), 200 (6), 400 (8) and 600 (12) 0 (14), 100 (3), 200 (6), 400 (8), 600 (12)	–
Fat suppression			
Philips	–	SPAIR/SPIR	–
Siemens	–	SPAIR	–

### Data analysis

The processing was performed fully automatically using QMRITools 4.2.9 (www.qmritools.com) in Mathematica 14.1 [20] according to Schlaffke et al. [15]. Subsequently, the data were visually inspected for abnormalities such as artifacts or muscle injuries.

For Philips raw data were available allowing offline reconstruction of fat and water maps. In detail, a principal component analysis (PCA) [21] was applied to denoise the Dixon and diffusion data, followed by calculating the signal-to-noise ratio (SNR). Based on the calculation of field maps for B0 and T2, the Dixon data were reconstructed using the Iterative decomposition of water and fat with echo asymmetry and least-squares estimation (IDEAL) method, which employs an iterative least-squares approach to accurately separate water and fat signals while correcting for phase shifts caused by B0 inhomogeneities [22]. For Siemens, no raw data were available, and the inline reconstructed water and fat maps from 6-point Dixon under XA30 Siemens software were used to calculate the relative fat fraction in percentage.

To determine wT2, the slice profile was calculated via Bloch simulation, followed by the application of an extended phase graph (EPG) fitting approach to get separate water and fat times [23]. The simulation of the T2 answer for different B1 values allows an accurate measurement of wT2, independent of B1 inhomogeneities. These data are then further processed and fitted voxel-wise via a dictionary method [24].

The diffusion data were preprocessed by performing motion and eddy current correction [25]. Subsequently, the anisotropic data were smoothed using an anisotropic filter [26, 27]. Intravoxel incoherent motion (IVIM) fitting was used to correct for pseudodiffusion processes for Philips. For Siemens, only high *b* values (*b* > 100) was used for IVIM correction. Tensors and the diffusion parameters fractional anisotropy (FA), mean diffusivity (MD), axial diffusivity (*λ*_1_), and radial diffusivity (RD) were calculated using weighted-linear-least-squares (WLLS) fitting [28, 29].

The segmentation of the 12 thigh muscles—biceps femoris (long and short head), semimembranosus, semitendinosus, adductor magnus, adductor longus, gracilis, vastus lateralis, vastus intermedius, vastus medialis, rectus femoris, and sartorius (Fig. 1)—was performed automatically using a UNET based on the out-phase Dixon images using QMRITools 4.1.2 (www.qmritools.com). The results were manually reviewed and refined in ITK-SNAP 3.8.0 (www.itksnap.org).

Whole volume tractography was conducted, and tracts within each muscle were selected based on the respective muscle segmentations. Following fiber tracking stop parameters were used: FA range 0.05–0.65, MD range 0.5–2.5 × 10^−3^ mm^2^/s, step size 2.25 mm, and maximum angle 30°. The segmented labels were used as masks to calculate values for each muscle and leg, and quality-check images were subsequently generated.

### Statistical analysis

The statistical analysis was performed using R 4.4.1 (www.r-project.org). To compare reproducibility, intraclass correlation coefficients (ICCs) by a two-way mixed-effects model for consistency were calculated collectively and separately for each muscle for the parameters FF, wT2, and the diffusion metrics, including FA, MD, *λ*_1_ and RD. Bland–Altman plots were generated for all parameters per muscle and across muscles and participants.

In addition, paired *t* tests for comparison of parameter means between scanners and Pearson correlation coefficients were computed and Q–Q plots of paired differences were used to assess normality. Correlation coefficients were interpreted as negligible (*r* < 0.3), low (*r* = 0.3–0.5) moderate (*r* = 0.5–0.7), high (*r* = 0.7–0.9), and very high (*r* > 0.9) [30]. Descriptive statistics, including mean and standard deviation (SD), were calculated. The within-subject coefficient of variation (wsCV) was computed as the mean of the individual coefficients of variation across subjects, which each subject’s CV defined as the SD divided by the mean of their repeated measures expressed as a percentage. The significance level for all tests was set to less than 0.05.

## Results

The study population included ten subjects (5 females) with a mean age of 27.2 ± 3.1 years, a BMI of 23.4 ± 3.5 kg/m^2^, and a height of 171.6 ± 7.1 cm.

A detailed, intra-individual description of demographic data, physical activities, mode and duration of transportation to the study site is provided in the supplements (Supplement Table S1).

**Fig. 2:**
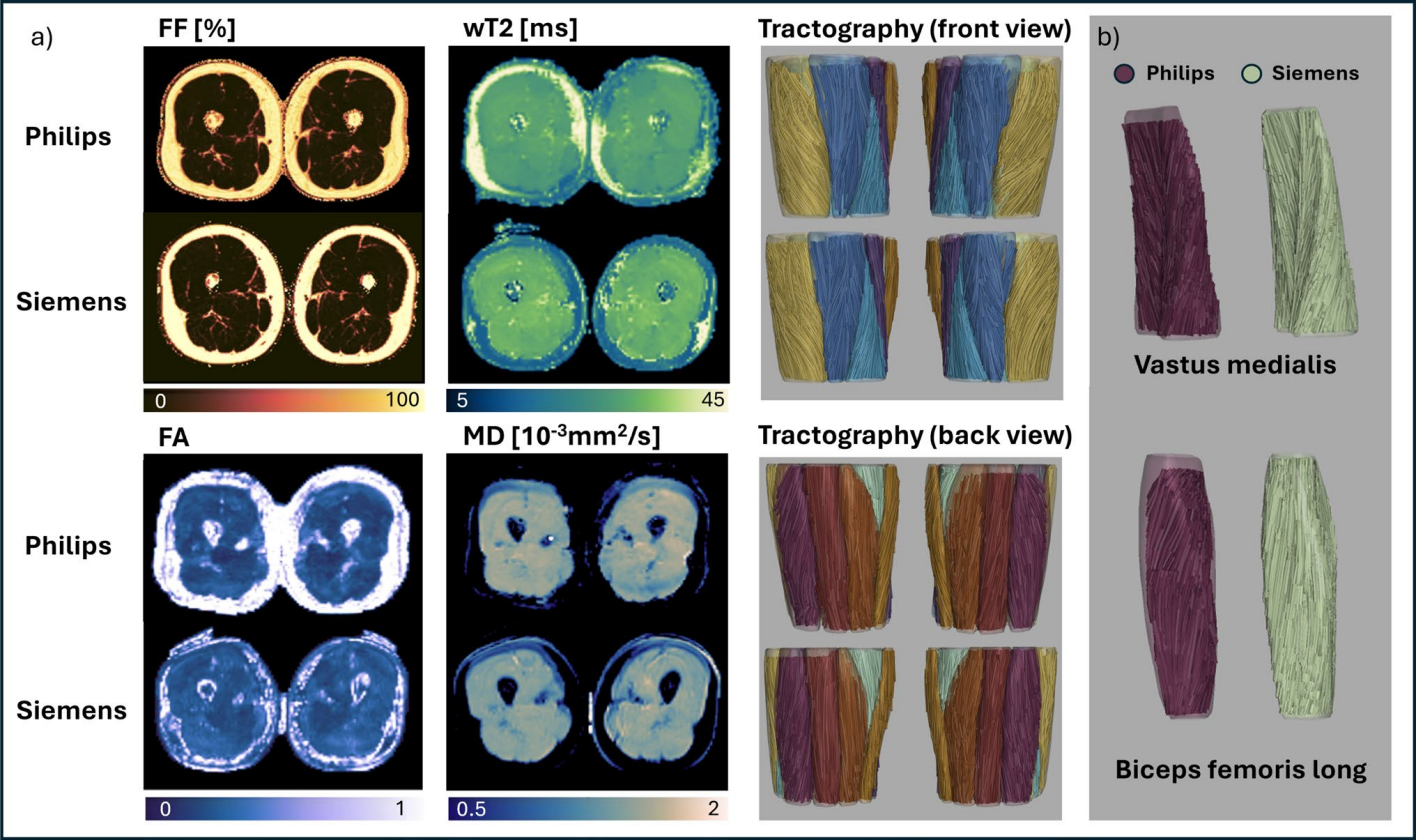
**a** Overview processed imaging data from a representative subject, showing quantitative MRI parameters, fat fraction (FF), water T2 relaxation time (wT2), fractional anisotropy (FA), mean diffusivity (MD) as well as diffusion tractography for upper leg muscles Color coding: yellow—vastus lateralis; dark blue—rectus femoris; light blue—vastus medialis; dark purple—gracilis; gold—sartorius; purple—biceps femoris long head; red—semitendinosus; orange—semimembranosus; turquoise—adductor magnus. **b** tractography of two representative muscles

All data were successfully acquired, except for one dataset per scanner due to data export failure. As a result, only nine complete datasets remained for each scanner. Since the excluded subjects differed between scanners, statistical analyses could only include eight matched subject pairs, corresponding to 384 muscles (8 participants × 12 muscles per leg × 2 legs). The remaining unmatched datasets, one per scanner, were complete but could not be used due to the lack of pairing. These datasets did not exhibit any outlying values and fell within the range of the included subjects (Supplement Table S2). Processed data from a representative subject are displayed in Fig. 2**,** and muscle-wise tractography results are presented in Supplementary (Supplement Fig. S3).

**Fig. 3:**
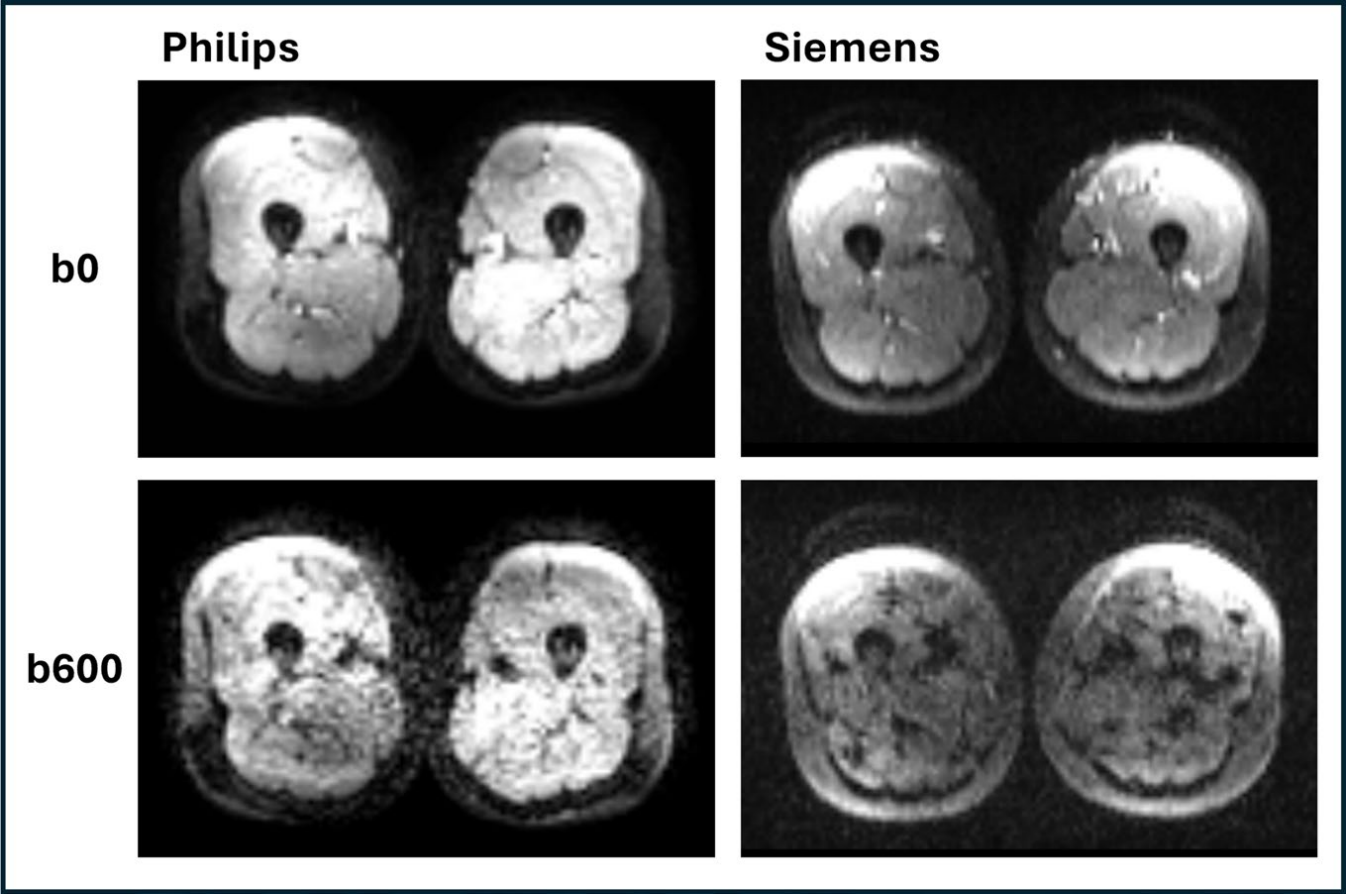
Raw diffusion-weighted images acquired on Philips and Siemens MRI systems at *b* values of 0 and 600

The datasets were suitable for analysis and exhibited adequate quality, enabling the successful execution of the automated processing. The SNR of the DTI data, calculated across all muscles and subjects, is 27.07 ± 3.48 for Philips and 21.08 ± 6.81 for Siemens, which is considered sufficient for analysis [31]. The average calculated B1 value for T2 calculation was 1.01 for Philips and 0.83 for Siemens. Representative raw diffusion-weighted images acquired on both MRI systems at *b* values of 0 and 600 s/mm^2^ are shown in Fig. 3.

The rectus femoris on the left side was excluded from the analysis of the diffusion parameters, as it was identified as an outlier in the Bland–Altman plots, which can be attributed to the coil configuration of the Philips MRI system resulting in low B1 and therefore low SNR [14].

**Fig. 4:**
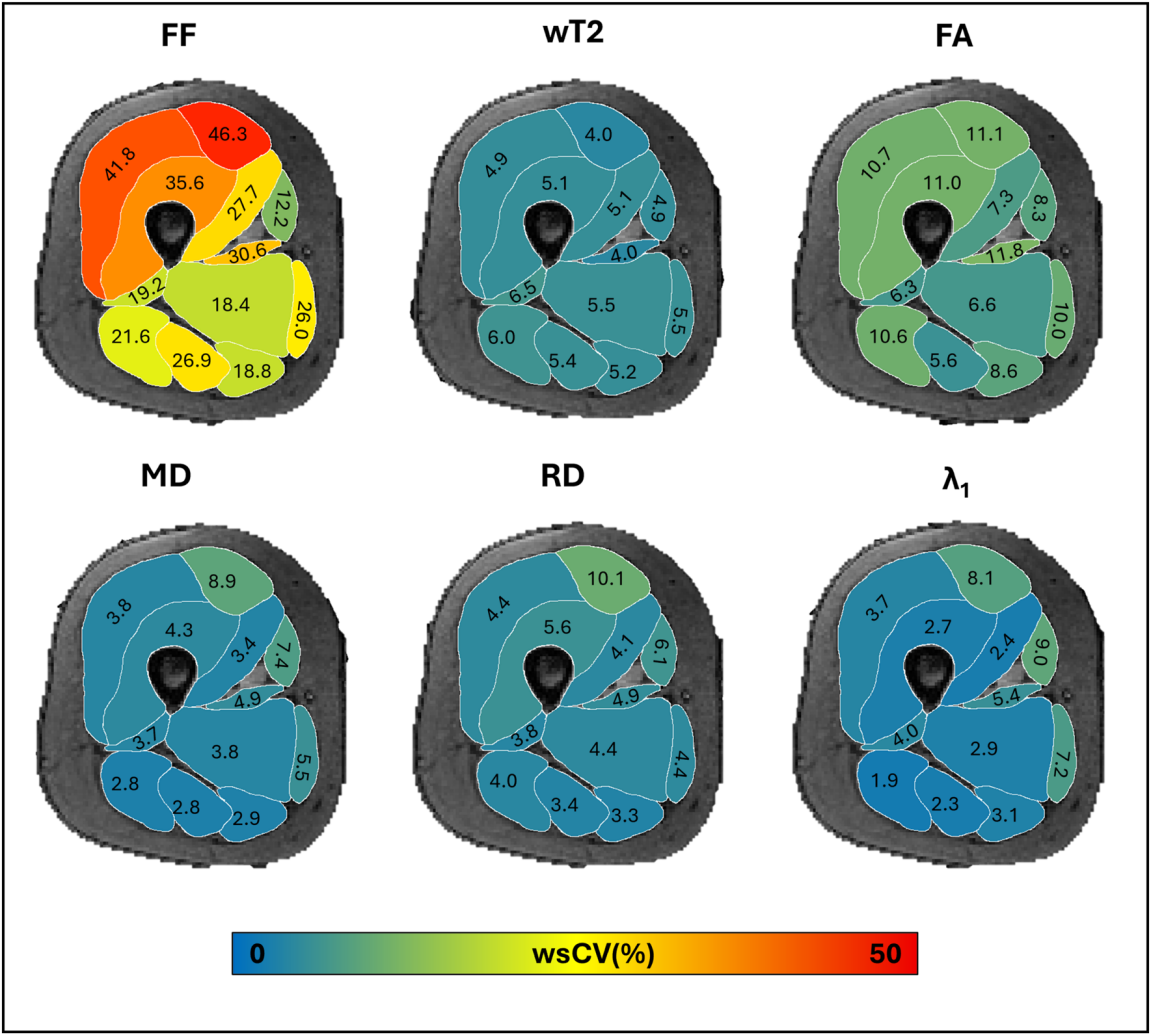
Within-subject coefficients of variation (in %) for fat fraction (FF), water T2 time (wT2), fractional anisotropy (FA), medial diffusivity (MD), radial diffusivity (RD) and axial diffusivity (*λ*_1_) across the 12 analyzed muscles

**Fig. 5:**
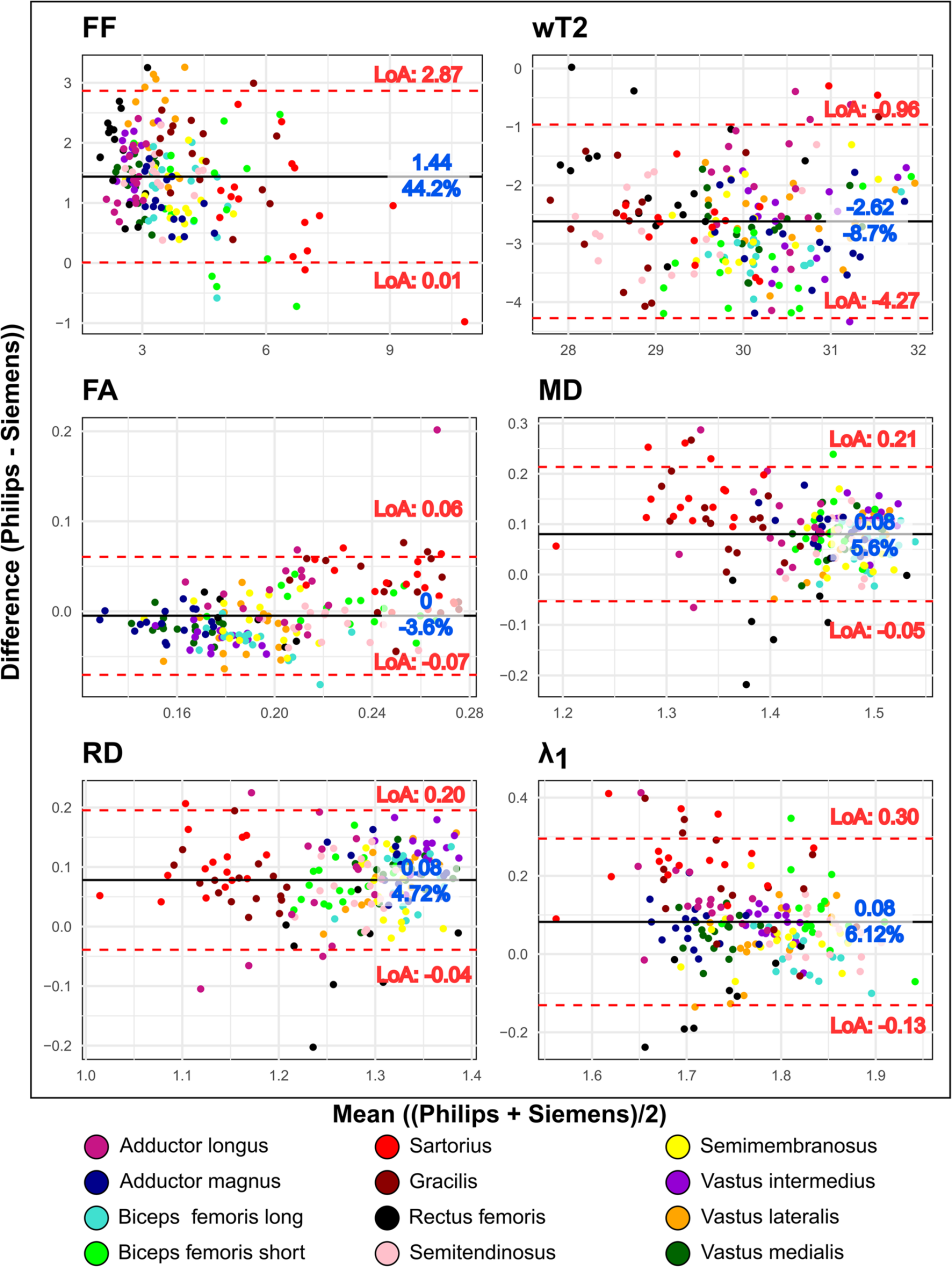
Bland–Altman Plots including the qMRI metrics FF, wT2, FA, MD, RD and *λ*_1_: All muscles, left and right side—*x*-axis shows the mean value from Philips and Siemens and *y*-axis the differences from Philips to Siemens for the qMRI parameters fat fraction (FF) in %, water T2 time (wT2) in ms, fractional anisotropy (FA), medial (MD), radial (RD) and axial (*λ*_1_) diffusivity in 10^–3^ mm^2^/s. The rectus femoris (left) was excluded for fractional anisotropy (FA) and mean diffusivity (MD). Red dotted lines indicate ± 1.96 standard deviations (95% confidence interval), and the black line shows the mean difference. The mean value is highlighted in blue

Descriptive analysis across all datasets and both vendors showed the following values: FF = 3.72 ± 1.54%, wT2 = 29.97 ± 1.66 ms, FA = 0.20 ± 0.04, MD = 1.44 × 10⁻^3^ mm^2^/s ± 0.08 × 10⁻^3^ mm^2^/s, RD = 1.28 ± 0.09 × 10⁻^3^ mm^2^/s and *λ*_1_ = 1.77 ± 0.10 × 10⁻^3^ mm^2^/s. A detailed vendor-specific descriptive analysis is summarized in Table 2, and a muscle-wise representation of the wsCVs is shown in Fig. 4, with wsCV ranging from 46.3 for FF in the rectus femoris to 1.9 for *λ*_1_ in the biceps femoris short head. Muscle- and vendor-wise descriptive parameters are provided in Supplement Table S4. Bland–Altman plots for all muscles are presented in Fig. 5, while individual muscle-specific plots are provided in the Supplement (Supplement Fig. S6), offering a visual representation of the reproducibility of measurements between vendors. Across all muscles, the mean bias ranged from 3.6% for FA to 44.2% for FF.

**Table 2:** Descriptive statistics including mean ± SD and within-subject CV for the qMRI metrics FF, wT2, FA, MD, RD and λ_1_

	Philips (*n* = 8)	Siemens (*n* = 8)	wsCV (%)
FF (%)	4.44 ± 1.23	3.01 ± 1.48	35.53
wT2 (ms)	28.66 ± 1.02	31.28 ± 1.01	5.16
FA	0.20 ± 0.04	0.20 ± 0.03	18.28
MD (10^−3^ mm^2^/s)	1.48 ± 0.06	1.40 ± 0.08	5.53
RD (10^−3^ mm^2^/s)	1.32 ± 0.09	1.24 ± 0.08	6.97
λ_1_ (10^−3^ mm^2^/s)	1.81 ± 0.07	1.73 ± 0.10	5.12

Mean values and standard deviation across all muscles for the following metrics: *FF* fat fraction, *wT2* water T2 relaxation time, *FA* fractional anisotropy, *MD* mean diffusivity, *RD* radial diffusivity, *λ*_*1*_ axial diffusivity, *qMRI* quantitative muscle magnetic resonance imaging

Pearson correlation coefficients revealed moderate to high correlation between vendors for FF (*r* = 0.87, *p* < 0.01), wT2 (*r* = 0.66, *p* < 0.01), FA (*r* = 0.67, *p* < 0.01), MD (*r* = 0.56, *p* < 0.01), and RD (*r* = 0.74, *p* < 0.01). In contrast, negligible correlations were observed for λ_1_ (*r* = 0.23, *p* < 0.05) [30]. Paired *t* tests revealed statistically significant differences between scanners for all parameters, with *p* values of 2.2 × 10⁻^1^⁶ for wT2, FF, MD, RD, λ_1_, and *p* = 0.048 for FA. Paired differences showed an approximate normal distribution based on Q–Q plots (Supplement Figure S5).

ICCs were excellent to moderate (FF = 0.923, wT2 = 0.792, FA = 0.777, MD = 0.708, RD = 0.850), except for *λ*_1_ (ICC = 0.361) [32]. In the muscle-wise analysis, the ICCs were notably lower (Supplement Table S5).

## Discussion

We evaluated the inter-vendor reproducibility of 3T MRI systems from Siemens and Philips in a traveling cohort of healthy adults. In the context of neuromuscular diseases and muscle injuries, such reproducibility is essential for reliable assessment of physiological and pathological changes as well as for comparability of data in research settings. Our findings demonstrate good reproducibility given by moderate to excellent ICCs (ICC = 0.708–0.923) and moderate to high significant correlations (*r* = 0.56–0.87) for the quantitative parameters FF, wT2, MD, FA, and RD, except for the eigenvector *λ*_1_ (ICC = 0.361; *r* = 023).

### Dixon-based fat quantification

The excellent inter-vendor reproducibility in FF (ICC > 0.9) was consistent with the findings of Eck et al., who reported similar inter-vendor reproducibility for proton density fat fraction (ICC = 0.92) across Philips and Siemens systems [17], despite using different acquisition methods (6-point and 4-point) in our study. We observed high wsCVs for FF, ranging from 12% in the Sartorius to 46% in the rectus femoris. Comparable CVs of 18.10% to 36.90% have been reported in prior studies assessing repeatability and reproducibility in different MRI systems in muscles with low fat content (< 5%) [33]. Notably, higher SNR recordings did not improve accuracy at small FF values, indicating a generally limited accuracy at small FF values [34]. The relatively high wsCVs in our study are attributable to the low absolute FF values, where even minor differences lead to disproportionately large variability. As CVs tend to be lower with higher fat fraction which has also been shown in a phantom study covering different FF ranges [35], and FF correlates more strongly with histopathology at higher fat levels [13], we propose that this effect is also relevant across MRI vendors. We therefore attribute the high variability observed in healthy muscle in our study primary to measurement variability in low FF, even though the CVs are higher in this study comparing to phantoms [35], reflecting the influence of using different vendors. The high variability in wsCV between muscles is consistent with different FF values between muscles and the increasing variability in lower FF values. In muscles with pathological fat replacement, lower wsCVs are expected, supporting the robustness of the method in clinical populations. Even with larger variation in a clinical population, accurate reproducibility can be assumed due to the high ICCs.

Despite the overall good reproducibility, we observed systematic, statistically significant differences between vendors. On average, FF values were 1.44% higher on Philips than on Siemens scanners. This observation aligns with previous findings from liver imaging, where Philips systems yielded FF values over 2% higher than Siemens and GE systems [36], and with phantom studies showing a difference of 2.39% between Philips and Siemens scanners [37]. Similar trends have been observed in skeletal muscle, with Siemens scanners typically reporting the lowest FF values [33]. These may be attributed to differences in Dixon implementations (4-point Dixon on Philips vs. 6-point on Siemens) and the used fat spectra reconstruction method [21]. Grimm et al. reported higher FF values when using 2- and 3-point Dixon compared to 6-point Dixon acquisitions [38], whereas Noble et al. found no consistent differences between 3- and 4-point Dixon methods [39]. Therefore, the higher FF values observed on the Philips scanner using a 4-point Dixon approach are in good agreement with previously reported findings. Additional contributing factors may include vendor-specific reconstruction algorithms or T1-related bias influenced by flip angle [39, 40]. However, given the relatively low flip angles used in our study (5° Siemens; 8° Philips), such an effect is likely minimal.

Finally, the intra-scanner precision of FF on Philips systems has previously been shown with limits of agreement (LoA) between 2.58% and 4.1% [41, 42]. Our findings fall within this range (2.88%), consistent with the limited accuracy and elevated wsCVs at low FF values discussed above [34].

### Water T2 relaxation time

For wT2, we observed a good reproducibility between vendors with an ICC of 0.792. While only a few studies have assessed the reproducibility of wT2 across vendors in skeletal muscle, Forbes et al. reported small inter-vendor wsCVs of ≤ 5.2% in leg muscles of two subjects [19, 43]. With wsCVs of 5.16%, our study now extends these findings by demonstrating high reproducibility for skeletal muscle in a larger cohort. Even higher inter-vendor reproducibility has already been demonstrated for the kidney in a study of eight subjects using an EPG-based model, showing an inter-vendor CV of < 3% [18].

Despite overall good reproducibility, a systematic offset in wT2 was observed, with values on average 2.62 ms (8.74%) higher on Siemens scanners.

This trend is consistent with previous studies showing Siemens-based wT2 to be approximately 1–2 ms higher [19, 43, 44]. A likely explanation lies in vendor-dependent B1 inhomogeneities, as described by Li et al. [18]. Lower B1 amplitudes can lead to an overestimation of wT2 due to stimulated echoes [45]. Despite accounting for these effects using EPG modeling and vendor-specific pulse profiles, which may reduce clinical applicability due to limited accessibility, residual discrepancies may persist. In line with this, the Siemens scanners in our study consistently exhibited lower B1 amplitudes than Philips, further supporting this hypothesis.

The LoA in our inter-vendor comparison was 5.23 ms, which is comparable to previously reported values of 2.5 ms for global T2 repeatability on Philips systems [46] and 5.6 ms for wT2 on Siemens systems [47]. This suggests that inter-vendor variability remains within an acceptable range, reinforcing the reliability of wT2 measurements across different MRI platforms.

### Diffusion metrics

Our study showed that the inter-vendor reproducibility for FA, MD, and RD was good (ICC > 0.708), but poor for *λ*_1_ (ICC = 0.361). Supporting this, the wsCVs for MD, RD, and *λ*_1_ were low between the vendors (< 7%). While Chianca et al. reported no significant inter-vendor differences in DWI parameters across GE, Siemens, and Philips systems [16], our data revealed statistically significant differences for all DWI metrics (*p* = 2.2 × 10⁻^1^⁶). FA showed a significant difference with a *p* value of 0.048. Despite these differences, the systematic inter-vendor shifts in MD and RD were minor (< 6.5%) and consistent with Chianca et al., who found shifts of approximately 0.05 between Philips and Siemens [16]. A potential explanation for these deviations is the difference in SNR, which was approximately 6 units lower on Siemens scanners but remained above an acceptable threshold (> 20) [31], potentially related to the higher acceleration factor used, or the higher *b* values used in Siemens. Since lower SNR can artificially lead to higher FA values [31, 48], this hypothesis is consistent with our results. It should be noted that, due to the use of higher *b* values (*b* > 100 s/mm^2^) in the Siemens compared to Philips multi-shell acquisition scheme, perfusion-related signal contributions may not be adequately corrected, and pseudodiffusion tends to overestimated in the absence of very low *b* values (< 50s/mm^2^) [49]. However, *b* values below 200 s/mm^2^ have been shown to improve the estimation of vascular diffusion effects [50].

Without IVIM correction, diffusion metrics (MD, AD, RD) positively correlate with the perfusion signal fraction. This nuisance effect can lead to increased variance within groups. Furthermore, MD is overestimated and FA underestimated, with these effects increasing at higher perfusion signal fraction values [51]. However, in our study, we observed opposite effects with higher MD values and lower FA values with Siemens. Overall, in our study of healthy muscle on the Siemens scanner, perfusion signal fraction is expected to be low and stable and the used *b* value of 100 s/mm^2^ seems to be sufficient, suggesting that the influence of the higher *b* values on perfusion-related bias is likely minor in our setting. Furthermore, potential chemical shift artifacts (Fig. 3), particularly in the anterior compartment muscles, were evaluated and did not show any noticeable impact in the muscle-wise analysis.

Compared to intra-scanner precision data, our DWI results show similar or even lower LoAs (LoA of FA = 0.11), whereas reported LoAs of FA are around 0.14 [42] or 0.3 [46]. These findings indicate that inter-vendor precision for FA between Siemens and Philips systems is not inferior to intra-scanner precision reported for Philips systems. Our analysis demonstrates comparable precision across vendor and within-vendor comparisons, suggesting that clinical interpretation is likely equally affected by measurement variability regardless of vendor. However, the relatively low precision overall may still limit the sensitivity to subtle or progressive pathological changes, as seen in slow-progressing NMDs.

### Implications for clinical practice

While high measurement precision is essential, clinical utility ultimately depends on whether quantitative differences reliably reflect pathological changes. In various NMDs, including Pompe disease [4], FSHD [5], or inclusion body myositis (IBM), the annual increase in FF is approximately 1–3%. These values are within the range of systematic bias and LoA found in our study. This may reduce the sensitivity to detect subtle disease progression, particularly in early or slowly progressive stages. While a 2% difference may be less relevant for diagnosis, it can play an important role in longitudinal assessment.

Disease-related elevations in wT2 are typically in the range between 2 and 5 ms in muscular dystrophies such as limb-girdle muscle dystrophy [52], FSHD [53], myotonic dystrophy type 1 [54], GNE-myopathy [55], and around 1–2 ms in conditions such as diabetic polyneuropathy [56] and spinal muscular atrophy [57]. Inflammatory myopathies as dermatomyositis and polymyositis show larger increases of about 15 ms in affected muscles or 3 ms in unaffected muscles [58]. Marathon-induced microtrauma is associated with changes of around 1 ms after 1–2 days [59]. These small differences highlight the need for high precision and minimal inter-vendor bias to ensure clinical utility. Our results indicate that differences between vendors are only partially, depending on underlying disease, sensitive to pathological changes in muscle tissue and should therefore be considered in clinical practice.

For DWI parameters, although MD and RD values remained within physiological reference ranges [60], the observed inter-vendor bias (MD and RD ~ 0.08 × 10⁻^3^ mm^2^/s) exceeds disease-related changes reported in inclusion body myositis and myotonic dystrophy (DM) or marathon-induced microtrauma (~ 0.03–0.07 × 10⁻^3^ mm^2^/s) [6, 8, 59]. In addition, the variability reported within pathological population, such as standard deviations of MD in DM (~ 0.05 × 10⁻^3^ mm^2^/s) [8], is smaller than the inter-vendor variability observed in our study (0.08 × 10⁻^3^ mm^2^/s), despite our cohort consisting of healthy subjects only. This suggests that the variability introduced by different vendors in their measurements may exceed the variability due to abnormality. Consequently, diagnostic sensitivity may be reduced in multi-vendor settings, where subtle pathological alterations may be obscured by systematic differences in measurement.

### Limitations

Several limitations should be acknowledged. This study focused on the reproducibility of qMRI parameters, while accuracy was not explicitly assessed. However, the obtained values are in line with previous reports [14, 60]. The inclusion of calibrated phantoms could further improve cross-vendor standardization. The small sample size (*n* = 10) of healthy adults may limit generalizability, particularly to populations with pathological conditions. Menstrual cycles and circadian rhythms, which may influence T2 signal intensity [61], were not tracked, since recording such parameters is not standard practice. The low number of data points challenges for muscle-specific analyses, particularly in the estimation of ICCs, and may account for the occurrence of negative ICC values observed exclusively in the muscle-wise evaluation [62]. The left rectus femoris was excluded due to its outlier status in the Philips dataset, a phenomenon previously observed by our group, possibly related to body coil cable positioning and B1⁺ inhomogeneities [14]. In line with previous studies reporting no significant side differences in DWI parameters [63], we did not differentiate between left and right sides. Nonetheless, a side-specific analysis could potentially uncover subtle effects. In addition, our analysis was limited to the mid-thigh region, which may fail to capture regional variations in qMRI values [59]. However, both aspects are of lesser relevance given the primary focus of this study on reproducibility. Although minor differences in acquisition protocols existed due to vendor-specific settings, key parameters (e.g., *b* values or echo train length) were kept constant to reduce bias.

## Conclusion

Our study demonstrates good inter-vendor reproducibility of qMRI parameters between 3T systems from Siemens and Philips in healthy skeletal muscle. However, switching MRI systems for the same subject can introduce problems, particularly in longitudinal studies. Although the parameters, including FF, wT2, MD, FA, and RD, showed vendor-related bias, the bias remained constant and may be addressed in linear mixed models, provided that between-scanner variance is sufficiently comparable. Systematic inter-vendor differences in absolute values were observed, particularly for FF and wT2, which must be considered when interpreting results across vendors. While reproducibility was good, the magnitude of bias and LoA may limit sensitivity for detecting subtle pathological changes, especially in slowly progressive neuromuscular diseases. The use of vendor-native acquisition protocols in this study provides realistic estimates of inter-vendor comparability under routine multi-center conditions. These findings underscore the importance of accounting for systematic bias in both clinical and research applications and highlight the need for harmonization strategies, such as standardized protocols or bias correction, to improve comparability in multi-center or longitudinal studies involving different MRI vendors. However, modifying vendor-specific sequences for harmonization may compromise clinical practicality. Effective bias correction requires an understanding of the underlying sources of bias, which may manifest differently in diseased muscle tissue. In addition, statistical harmonization approaches such as ComBat have shown promise in reducing scanner-related bias in multi-center quantitative MRI studies in the brain [64] and may provide a complementary solution when full acquisition harmonization is impractical. Future work should establish bias-correction models and harmonized acquisition protocols across vendors to enable quantitative pooling in multi-site trials.

## Supplementary Information

Below is the link to the electronic supplementary material.

Supplementary file1 (DOCX 3060 KB)

## Data Availability

The data that support the findings of this study are available from the corresponding author, Lara Schlaffke, upon reasonable request.

## Abbreviations

DTI: Diffusion tensor imaging
DM: Myotonic dystrophy
EPG: Extended phase graph
FA: Fractional anisotropy
FF: Fat fraction
FOV: Field of view
FSHD: Facioscapulohumeral muscle dystrophy
ICC: Intraclass correlation coefficient
IDEAL: Iterative decomposition of water and fat with echo asymmetry and least-squares estimation
IVIM: Intravoxel incoherent motion
LoA: Limits of agreement
MD: Mean diffusivity
MESE: Multi-echo spin-echo sequence
NMD: Neuromuscular diseases
PCA: Principal component analysis
qMRI: Quantitative muscle imaging
RD: Radial diffusivity
SE-EPI: Spin-echo echo-planar sequence
SNR: Signal to noise ratio
WLLS: Weighted linear least squares
wsCV: Within-subject coefficient of variation
wT2: Water T2 relaxation time
λ_1_: Axial diffusivity
