# Supplementary material for: Standardizing quantitative muscle MRI: an inter-vendor evaluation in healthy adults
Source: MAGMA. 2026 Feb 12;39(4):631–43. doi: 10.1007/s10334-026-01328-9 (PMC13447357; doi:10.1007/s10334-026-01328-9)
Supplement: Supplementary file 1 [file 10334_2026_1328_MOESM1_ESM.docx]

**Standardizing Quantitative Muscle MRI: An Inter-Vendor Evaluation in Healthy Adults**

Johanna Thomä^1,2^, Lionel Butry^1,2^, Martijn Froeling^3^, Marco Meixner^4,7^, Johannes Forsting^[1],2^, and Lara Schlaffke^1,2,5,6^

*^1^Department of Neurology, BG-University Hospital Bergmannsheil, Ruhr-University Bochum, 44789, Bochum, Germany*

*^2^Heimer Institute for Muscle Research, BG-University Hospital Bergmannsheil, Ruhr-University Bochum, 44789, Bochum, Germany*

*^3^Center for Image Sciences, Precision Imaging Group, Division Imaging & Oncology, University Medical Centre Utrecht, Utrecht, Netherlands*

*^4^Institute of Neuroradiology, St. Josef Hospital, Ruhr-University Bochum, 44791 Bochum, Germany*

*^5^Dep. Information Technology, University of Applied Sciences and Arts, 44139, Dortmund, Germany*

*^6^University Hospital of Pediatrics and Adolescent Medicine, Ruhr-University Bochum, 44791, Bochum, Germany*

*^7^Department of Neurology, St. Josef Hospital, Ruhr-University Bochum, 44791, Bochum, Germany*

Prospective Journal:	Magnetic Resonance Materials in Physics, Biology and Medicine

Corresponding author:	Lara Schlaffke

Department of Neurology,

BG University Hospital Bergmannsheil,

Ruhr-University Bochum,

Bürkle-de-la-Camp-Platz 1,

44789 Bochum, Germany

Lara.Schlaffke@ruhr-uni-bochum.de

+49 234 302 6810

**Supplements:**

**Table S1: Demographic Data and Self-Reported Physical Activity (Intra-individual)**
Overview of age, body mass index (BMI), sex, travel mode and duration to each MRI scanner location (Philips and Siemens), and self-reported time since last physical activity prior to each scan. The same individuals underwent imaging on both systems.

| **Subject ID** | **Age** | **BMI** | **Sex (f/m)** | **Travel to Philips (duration in min)** | **Travel to Siemens (duration in min)** | **Days since last activity (Philips)** | **Days since last activity (Siemens)** |
| --- | --- | --- | --- | --- | --- | --- | --- |
| CON_01 | 23 | 22.50 | f | Active (13) | Active (7) | – | – |
| CON_02 | 33 | 23.24 | m | Active (7) | Active (5) | – | – |
| CON_03 | 27 | 20.76 | f | Passive | Active (30) | – | 2 |
| CON_04 | 24 | 28.41 | m | Passive | Active (30) | – | 1 |
| CON_05 | 30 | 25.47 | m | Passive | Passive | 5 | – |
| CON_06 | 28 | 28.73 | f | Passive | Passive | – | 3 |
| CON_07 | 28 | 21.88 | f | Active (15) | Active (20) | – | – |
| CON_08 | 29 | 24.28 | m | Passive | Passive | – | – |
| CON_09 | 24 | 17.65 | f | Passive | Passive | – | – |
| CON_10 | 26 | 21.01 | m | Active (8) | Active (15) | – | 1 |

**Note:** Sex is abbreviated as f = female, m = male.
Travel mode was grouped as "Active" (walking or cycling) and "Passive" (car or public transport).
"Days since last activity" refers to the number of days between the last reported physical activity and the respective MRI session.
“–” indicates no activity reported.

**Table S2: Excluded datasets:** Overview of the two excluded datasets. Both subjects had complete data for one scanner but were excluded due to the absence of a matching dataset from the other scanner. Parameter values were within the range of the included subjects.

**Philips: Subject Nr. 09 (female):**

| **Muscle** | **Side** | **FF** | **wT2** | **FA** | **MD** | **RD** | **λ1** |
| --- | --- | --- | --- | --- | --- | --- | --- |
| Biceps femoris long | Left | 3.8713 | 29.0403 | 0.2199 | 1.4701 | 1.2852 | 1.8419 |
| Biceps femoris long | Right | 3.4838 | 28.9275 | 0.2092 | 1.4728 | 1.2983 | 1.8312 |
| Biceps femoris short | Left | 3.5932 | 28.1704 | 0.2448 | 1.4194 | 1.2213 | 1.8094 |
| Biceps femoris short | Right | 3.2906 | 28.4557 | 0.2494 | 1.4263 | 1.2178 | 1.8304 |
| Semimembranosus | Left | 3.5107 | 29.2568 | 0.2029 | 1.465 | 1.2951 | 1.8004 |
| Semimembranosus | Right | 3.5384 | 29.0634 | 0.2198 | 1.478 | 1.2923 | 1.8457 |
| Semitendinosus | Left | 3.6258 | 28.0357 | 0.2400 | 1.4678 | 1.263 | 1.8739 |
| Semitendinosus | Right | 3.6416 | 28.0552 | 0.2730 | 1.4591 | 1.2285 | 1.9200 |
| Adductor magnus | Left | 3.7683 | 28.9107 | 0.1932 | 1.3984 | 1.2427 | 1.7025 |
| Adductor magnus | Right | 3.5744 | 29.2943 | 0.1723 | 1.4509 | 1.3079 | 1.7340 |
| Adductor longus | Left | 3.2623 | 29.0014 | 0.2363 | 1.3518 | 1.1713 | 1.7124 |
| Adductor longus | Right | 2.7947 | 28.6392 | 0.2484 | 1.4043 | 1.2055 | 1.7868 |
| Gracilis | Left | 5.1712 | 28.0431 | 0.3373 | 1.3549 | 1.093 | 1.9060 |
| Gracilis | Right | 5.5438 | 28.2235 | 0.3283 | 1.3329 | 1.0689 | 1.8575 |
| Vastus lateralis | Left | 3.9436 | 29.4672 | 0.2017 | 1.4419 | 1.2712 | 1.7757 |
| Vastus lateralis | Right | 3.8194 | 29.7598 | 0.2197 | 1.4787 | 1.2844 | 1.8585 |
| Vastus intermedius | Left | 3.6854 | 29.5119 | 0.1865 | 1.4708 | 1.3178 | 1.7784 |
| Vastus intermedius | Right | 3.336 | 29.63 | 0.1822 | 1.4984 | 1.3463 | 1.8003 |
| Vastus medialis | Left | 3.3678 | 29.1958 | 0.1879 | 1.4179 | 1.2652 | 1.7122 |
| Vastus medialis | Right | 3.1441 | 29.2013 | 0.1892 | 1.4324 | 1.2713 | 1.7404 |
| Rectus femoris | Left | 3.4458 | 28.3106 | 0.2461 | 1.2376 | 1.0445 | 1.6037 |
| Rectus femoris | Right | 3.148 | 28.5382 | 0.2299 | 1.0337 | 0.8977 | 1.3216 |
| Sartorius | Left | 5.2048 | 28.2972 | 0.2903 | 1.2089 | 1.0056 | 1.6194 |
| Sartorius | Right | 5.7905 | 28.589 | 0.2991 | 1.2549 | 1.0303 | 1.6871 |
| **Mean** |  | **3.8148** | **28.8174** | **0.2336** | **1.3928** | **1.2052** | **1.7646** |
| **SD** |  | 0.7867 | 0.5402 | 0.0452 | 0.1107 | 0.1182 | 0.1252 |

**Siemens: Subject Nr. 10 (male):**

| **Muscle** | **Side** | **FF** | **wT2** | **FA** | **MD** | **RD** | **λ1** |
| --- | --- | --- | --- | --- | --- | --- | --- |
| Biceps femoris long | Left | 2.2000 | 31.1100 | 0.2127 | 1.4740 | 1.2913 | 1.8427 |
| Biceps femoris long | Right | 2.2000 | 31.0871 | 0.2155 | 1.4838 | 1.2994 | 1.8523 |
| Biceps femoris short | Left | 2.3000 | 30.5713 | 0.2766 | 1.4257 | 1.1954 | 1.8843 |
| Biceps femoris short | Right | 2.4000 | 30.8565 | 0.2555 | 1.4393 | 1.2242 | 1.8706 |
| Semimembranosus | Left | 2.2000 | 31.0633 | 0.2179 | 1.4677 | 1.2845 | 1.8422 |
| Semimembranosus | Right | 2.1000 | 31.4747 | 0.2132 | 1.4708 | 1.2893 | 1.8377 |
| Semitendinosus | Left | 1.7000 | 29.9232 | 0.2655 | 1.4466 | 1.2208 | 1.8988 |
| Semitendinosus | Right | 1.7000 | 30.6895 | 0.2736 | 1.4411 | 1.2077 | 1.9090 |
| Adductor magnus | Left | 2.0000 | 32.1899 | 0.1660 | 1.4372 | 1.3010 | 1.7050 |
| Adductor magnus | Right | 1.9000 | 32.8414 | 0.1656 | 1.4461 | 1.3105 | 1.7178 |
| Adductor longus | Left | 1.6000 | 31.4807 | 0.2060 | 1.3862 | 1.2226 | 1.7098 |
| Adductor longus | Right | 1.6000 | 30.9114 | 0.2592 | 1.3543 | 1.1520 | 1.7473 |
| Gracilis | Left | 1.9500 | 30.8154 | 0.2810 | 1.3405 | 1.1171 | 1.7874 |
| Gracilis | Right | 1.7000 | 30.4638 | 0.2739 | 1.3598 | 1.1363 | 1.7961 |
| Vastus lateralis | Left | 1.8000 | 32.1497 | 0.2011 | 1.4721 | 1.3030 | 1.8045 |
| Vastus lateralis | Right | 1.8000 | 31.3971 | 0.2034 | 1.4530 | 1.2835 | 1.7880 |
| Vastus intermedius | Left | 1.7000 | 32.2242 | 0.1920 | 1.4716 | 1.3103 | 1.7855 |
| Vastus intermedius | Right | 1.8000 | 31.8802 | 0.1900 | 1.4636 | 1.3043 | 1.7753 |
| Vastus medialis | Left | 1.9000 | 31.7396 | 0.1670 | 1.4483 | 1.3098 | 1.7210 |
| Vastus medialis | Right | 2.2000 | 31.7199 | 0.1766 | 1.4420 | 1.2959 | 1.7306 |
| Rectus femoris | Left | 1.6000 | 30.1387 | 0.1951 | 1.4169 | 1.2535 | 1.7364 |
| Rectus femoris | Right | 2.4000 | 29.8352 | 0.2046 | 1.4254 | 1.2493 | 1.7528 |
| Sartorius | Left | 2.7000 | 29.7833 | 0.2719 | 1.2962 | 1.0887 | 1.7113 |
| Sartorius | Right | 2.6000 | 30.1783 | 0.2829 | 1.3055 | 1.0862 | 1.7349 |
| **Mean** |  | **2.0021** | **31.1052** | **0.2236** | **1.4237** | **1.2390** | **1.7892** |
| **SD** |  | 3.2386 | 0.8310 | 0.0406 | 0.0542 | 0.0742 | 0.0646 |

**Figure S3: Tractography data:** Tractography results of a representative subject for each investigated muscle comparing both MRI scanners.

**Table S4: Muscle-wise and vendor-wise descriptive analyses:** mean values and standard derivation (SD) for each muscle and parameter sorted by manufacturer and calculated from the 8 included subjects

**Philips**

| **Muscle** | **FF** | | **wT2** | | **FA** | | **MD** | | **RD** | | **λ_1_** | | **λ_2_** | | **λ_3_** | |  |
| --- | --- | --- | --- | --- | --- | --- | --- | --- | --- | --- | --- | --- | --- | --- | --- | --- | --- |
|  | mean | ±SD | mean | ±SD | mean | ±SD | mean | ±SD | mean | ±SD | mean | ±SD | mean | ±SD | mean | ±SD | |
| Adductor longus | 3.4250 | 0.4446 | 29.3420 | 0.7353 | 0.2182 | 0.0451 | 1.4508 | 0.0612 | 1.2753 | 0.0820 | 1.7869 | 0.0452 | 1.3598 | 0.0673 | 1.1910 | 0.0946 | |
| Adductor magnus | 4.0968 | 0.4485 | 29.1674 | 0.7216 | 0.1497 | 0.0158 | 1.4988 | 0.0241 | 1.3735 | 0.0288 | 1.7445 | 0.0364 | 1.4260 | 0.0290 | 1.3207 | 0.0290 | |
| Biceps femoris long | 4.5207 | 0.6413 | 28.8392 | 0.7613 | 0.1747 | 0.0148 | 1.5261 | 0.0364 | 1.3741 | 0.0409 | 1.8264 | 0.0306 | 1.4249 | 0.0400 | 1.3234 | 0.0423 | |
| Biceps femoris short | 5.1764 | 1.0681 | 28.4731 | 1.0214 | 0.2328 | 0.0247 | 1.5010 | 0.0353 | 1.3014 | 0.0433 | 1.8938 | 0.0490 | 1.3714 | 0.0438 | 1.2293 | 0.0467 | |
| Gracilis | 5.4383 | 0.9686 | 27.5591 | 1.1593 | 0.2640 | 0.0229 | 1.4132 | 0.0362 | 1.1984 | 0.0346 | 1.8375 | 0.0628 | 1.2598 | 0.0320 | 1.1363 | 0.0382 | |
| Rectus femoris | 3.3810 | 0.4731 | 28.0230 | 0.7603 | 0.2047 | 0.0375 | 1.3060 | 0.1917 | 1.1517 | 0.1881 | 1.6095 | 0.2146 | 1.2107 | 0.1795 | 1.0938 | 0.1996 | |
| Sartorius | 7.0540 | 1.3801 | 28.4459 | 1.1234 | 0.2657 | 0.0184 | 1.3981 | 0.0620 | 1.1793 | 0.0542 | 1.8230 | 0.0833 | 1.2481 | 0.0570 | 1.1090 | 0.0524 | |
| Semimembranosus | 4.4261 | 0.6045 | 28.7398 | 0.8211 | 0.1848 | 0.0193 | 1.5047 | 0.0287 | 1.3441 | 0.0230 | 1.8189 | 0.0621 | 1.4008 | 0.0239 | 1.2873 | 0.0238 | |
| Semitendinosus | 4.1079 | 0.5844 | 27.8208 | 0.9807 | 0.2160 | 0.0190 | 1.4996 | 0.0331 | 1.3128 | 0.0368 | 1.8761 | 0.0408 | 1.3550 | 0.0324 | 1.2694 | 0.0420 | |
| Vastus intermedius | 3.6863 | 0.4253 | 29.3372 | 0.7173 | 0.1625 | 0.0110 | 1.5530 | 0.0235 | 1.4132 | 0.0288 | 1.8311 | 0.0211 | 1.4690 | 0.0286 | 1.3563 | 0.0326 | |
| Vastus lateralis | 4.4401 | 0.5127 | 29.3213 | 0.7660 | 0.1764 | 0.0211 | 1.5159 | 0.0564 | 1.3641 | 0.0569 | 1.8038 | 0.0839 | 1.4167 | 0.0564 | 1.3095 | 0.0618 | |
| Vastus medialis | 3.5625 | 0.3370 | 28.8652 | 0.6107 | 0.1529 | 0.0103 | 1.5050 | 0.0366 | 1.3747 | 0.0406 | 1.7565 | 0.0342 | 1.4238 | 0.0419 | 1.3218 | 0.0410 | |

**Siemens**

| **Muscle** | **FF** | | **wT2** | | **FA** | | **MD** | | **RD** | | **λ_1_** | | **λ_2_** | | **λ_3_** | |  |
| --- | --- | --- | --- | --- | --- | --- | --- | --- | --- | --- | --- | --- | --- | --- | --- | --- | --- |
|  | mean | ±SD | mean | ±SD | mean | ±SD | mean | ±SD | mean | ±SD | mean | ±SD | mean | ±SD | mean | ±SD | |
| Adductor longus | 2.0031 | 2.6986 | 31.3196 | 0.6518 | 0.1895 | 0.0217 | 1.3570 | 0.0609 | 1.2116 | 0.0599 | 1.6440 | 0.0749 | 1.2645 | 0.0663 | 1.1597 | 0.0580 | |
| Adductor magnus | 3.0500 | 6.9952 | 32.0549 | 0.7670 | 0.1621 | 0.0161 | 1.4105 | 0.0437 | 1.2797 | 0.0480 | 1.6710 | 0.0460 | 1.3193 | 0.0518 | 1.2402 | 0.0455 | |
| Biceps femoris long | 3.3563 | 8.6716 | 31.9670 | 0.5074 | 0.2079 | 0.0226 | 1.4704 | 0.0278 | 1.2920 | 0.0289 | 1.8219 | 0.0540 | 1.3344 | 0.0278 | 1.2490 | 0.0306 | |
| Biceps femoris short | 4.1094 | 13.5329 | 31.8226 | 0.6555 | 0.2283 | 0.0299 | 1.4134 | 0.0310 | 1.2248 | 0.0288 | 1.7878 | 0.0769 | 1.2671 | 0.0310 | 1.1825 | 0.0304 | |
| Gracilis | 3.6125 | 11.2953 | 30.0992 | 0.8191 | 0.2342 | 0.0298 | 1.3040 | 0.0659 | 1.1238 | 0.0505 | 1.6578 | 0.1123 | 1.1570 | 0.0509 | 1.0901 | 0.0495 | |
| Rectus femoris | 1.5313 | 5.5464 | 29.8864 | 0.9722 | 0.1966 | 0.0224 | 1.4363 | 0.0578 | 1.2710 | 0.0697 | 1.7598 | 0.0469 | 1.3136 | 0.0666 | 1.2273 | 0.0738 | |
| Sartorius | 6.1000 | 18.8361 | 30.8730 | 0.7005 | 0.2339 | 0.0264 | 1.2454 | 0.0480 | 1.0756 | 0.0425 | 1.5794 | 0.0770 | 1.1129 | 0.0442 | 1.0378 | 0.0420 | |
| Semimembranosus | 3.3063 | 6.7178 | 31.3811 | 0.5357 | 0.1964 | 0.0235 | 1.4512 | 0.0410 | 1.2857 | 0.0420 | 1.7785 | 0.0633 | 1.3247 | 0.0424 | 1.2456 | 0.0431 | |
| Semitendinosus | 2.6500 | 6.3875 | 30.4824 | 0.7565 | 0.2320 | 0.0224 | 1.4434 | 0.0307 | 1.2467 | 0.0372 | 1.8298 | 0.0494 | 1.2874 | 0.0377 | 0.1206 | 0.0386 | |
| Vastus intermedius | 1.9875 | 3.1596 | 32.0322 | 0.6804 | 0.1932 | 0.0154 | 1.4443 | 0.0236 | 1.2843 | 0.0268 | 1.7578 | 0.0370 | 1.3316 | 0.0233 | 1.2353 | 0.0316 | |
| Vastus lateralis | 2.1438 | 5.1765 | 31.8728 | 0.7896 | 0.1987 | 0.0175 | 1.4428 | 0.0188 | 1.2784 | 0.0234 | 1.7672 | 0.0346 | 1.3296 | 0.0209 | 1.2259 | 0.0314 | |
| Vastus medialis | 2.2125 | 3.9644 | 31.5480 | 0.6263 | 0.1712 | 0.0147 | 1.4261 | 0.0266 | 1.2854 | 0.0305 | 1.7047 | 0.0314 | 1.3227 | 0.0287 | 1.2466 | 0.0342 | |

**Table S5: Muscle-wise ICCs:** Intraclass correlation coefficients (ICCs) calculated for each muscle separately. Data are based on 8 subjects, with left and right sides analysed together resulting in a total of 16 data points per muscle

| **Muscle** | **FF** | **wT2** | **FA** | **MD** | **RD** | **λ_1_** |
| --- | --- | --- | --- | --- | --- | --- |
| Vastus lateralis | 0.379 | 0.830 | 0.066 | 0.366 | 0.585 | -0.138 |
| Vastus medialis | 0.525 | 0.774 | 0.754 | 0.380 | 0.629 | -0.110 |
| Vastus intermedius | 0.395 | 0.548 | 0.802 | -0.084 | 0.512 | -0.303 |
| Rectus femoris | -0.163 | 0.659 | 0.680 | 0.403 | 0.531 | 0.150 |
| Biceps femoris long | 0.771 | 0.880 | 0.726 | 0.091 | 0.432 | 0.331 |
| Biceps femoris short | 0.806 | 0.784 | 0.851 | -1.027 | 0.632 | -0.096 |
| Semimembranosus | 0.864 | 0.703 | 0.564 | 0.059 | -0.584 | 0.607 |
| Semitendinosus | 0.870 | 0.808 | 0.844 | -0.292 | 0.300 | 0.465 |
| Adductor magnus | 0.851 | 0.743 | 0.759 | 0.558 | 0.704 | 0.238 |
| Adductor longus | 0.136 | -0.368 | -0.265 | 0.138 | 0.259 | -0.323 |
| Gracilis | 0.891 | 0.699 | 0.448 | 0.288 | 0.648 | 0.193 |
| Sartorius | 0.920 | 0.647 | 0.691 | 0.626 | 0.710 | 0.624 |

**Figure S5: Q-Q Plots of Paired Differences for Normality Assessment**

**Figure S6:** **Bland–Altman plots for all qMRI parameters for the 12 thigh muscles**
Each point shows the difference vs. mean of Siemens and Philips measurements per subject and side for every muscle. Black line: mean difference; red dashed lines: 95% limits of agreement. Colours = subjects, shapes = left/right.
